# Covid-19, leukemia, and secondary malignancies of the skin – is there a connection: a case report and literature analysis

**DOI:** 10.3389/fonc.2023.1265479

**Published:** 2023-10-27

**Authors:** Olga Bogomolets, Ewa Rojczyk, Roman Hryshchenko, Catherine Bogomolets, Oleksandr Berezkin

**Affiliations:** ^1^Faculty of Medicine, Academy of Silesia in Katowice, Zabrze, Poland; ^2^Dermatology Department, Bogomolets Clinic, Kyiv, Lviv, Ukraine; ^3^Bogomolets Medical Laboratories, DERMPATHLab, Kyiv, Lviv, Ukraine

**Keywords:** Gleevec, chronic myeloid leukemia, melanoma, basal cell carcinoma, Covid-19

## Abstract

We report the case of a patient who was diagnosed with two melanomas and one skin cancer within two years. Of particular interest was the fact, that at the time these tumors were diagnosed, the patient was already suffering from chronic myeloid leukemia, which developed three months after recovering from Covid-19. From the time of leukemia occurrence, the patient has been taking the tyrosine kinase inhibitor (TKI) - Gleevec. Thus, we took into the account the possibile effect of Gleevec administration on the risk of skin tumor appearance. It was also important to analyze the impact of the SARS-CoV-2 virus and chronic myeloid leukemia on the risk of secondary malignancies. According to so far published data, the direct relationship between Gleevec treatment and the occurrence of skin cancers cannot be proved. However, literature data indicate a direct and indirect relationship between SARS-CoV-2 infection and an increased incidence of carcinogenesis.

## 1 Introduction

According to the World Health Organization (WHO) statistics, cancer is a leading cause of death worldwide, accounting for nearly 10 million deaths in 2020, that is about one in six deaths in general (1). In 2020, сhronic myeloid leukemia (CML) was diagnosed 474,519 times (2.6% of all diagnosed cancers), whereas melanoma of the skin is the 17th most common cancer worldwide – it was diagnosed 324,635 times (1.8% of cancers). Non-melanoma skin cancers (like basal cell carcinoma and squamous cell carcinoma) are often not fully recorded by cancer registries - registrations of these cancers are often incomplete, because most cases are successfully treated via surgery or ablation (2). That is why the reported global incidence of skin cancers is possibly underestimated.

At the same time, it’s very important to register all cases of melanoma and non-melanoma, especially because they may be associated (just like other types of cancer) with Covid-19 pandemic (3, 4). Indeed, Costa et al. presented a theoretical framework describing how Covid-19 could influence the development of blood cancers (3). Another study suggested that an abnormal immune response to viral infections can indirectly trigger leukemia promoting gene mutations and that SARS-CoV-2 can also significantly interact with the renin-angiotensin system, which is believed to play a role in the development of cancerous blood cells (5).

Some researchers also report the possible carcinogenic effect of the drug used in the treatment of leukemia - Imatinib mesylate (Gleevec) (6) and the occurrence of other types of cancer in the background of already existing chronic leukemia (7). It is also known, that Covid-19 alone or even SARS-CoV-2 vaccine booster dose can trigger non-melanoma skin cancer often preceded by multiple cutaneous adverse reactions (8, 9).

The aim of our study is to analyze the available data concerning the possibility of the influence of CML and the drug Gleevec or its analogs (which are used as the drug of choice in the treatment of leukemia) on the risk of future skin cancer developmend. Additionally, since our patient recoverd from Covid-19 before the onset of leukemia and skin neoplasms, the study also focused on the analysis of the potential effect of a previously transmitted viral disease (in particular - Covid-19), on the likelihood of cancer development.

## 2 Case description

**Figure 1:**
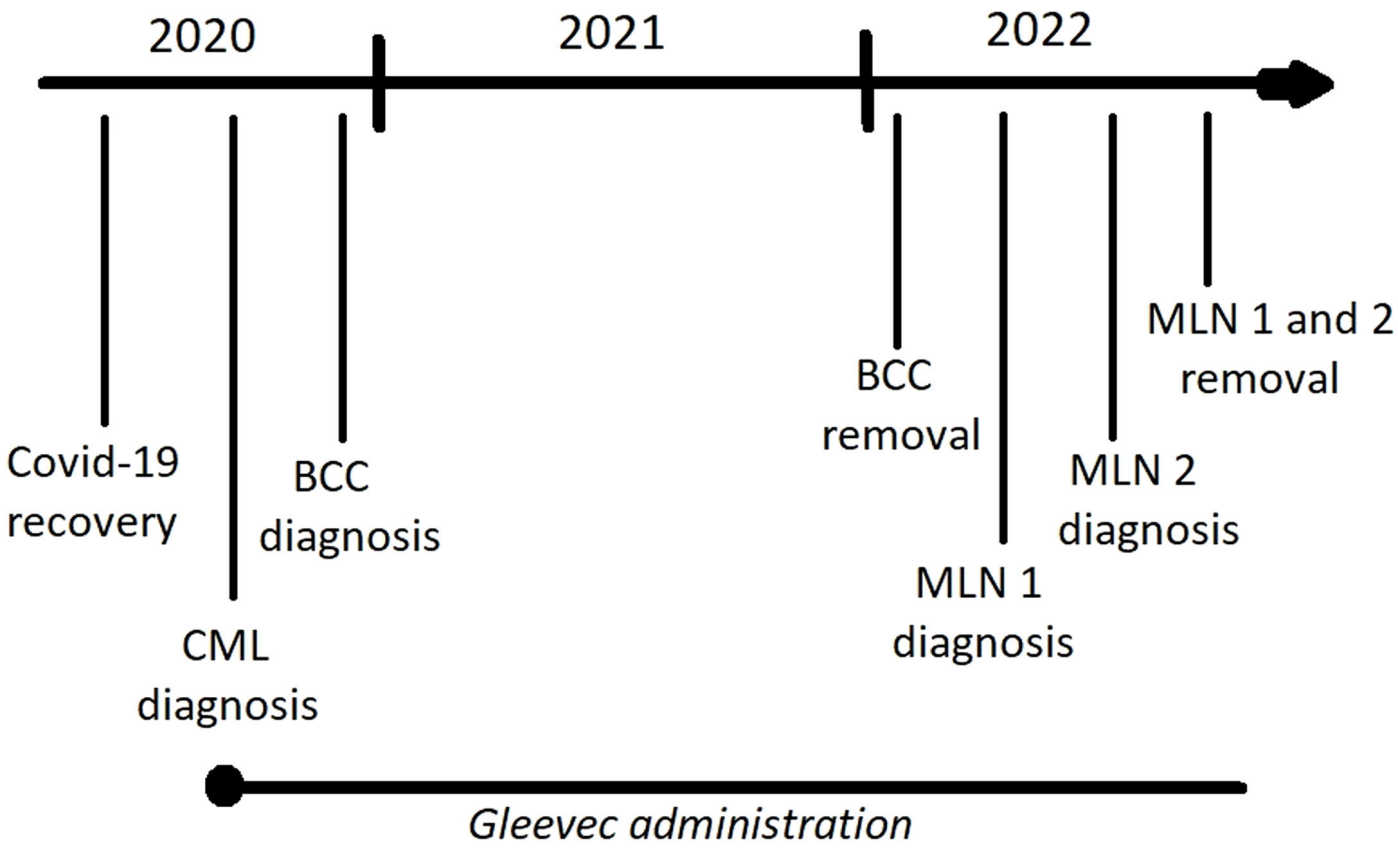
Timeline showing the sequence of disease occurrence and main treatment strategies (Gleevec administration, lesion surgical removal); BCC – basal cell carcinoma (neck), MLN 1 – the first melanoma (nose), MLN 2 – the second melanoma (back).

We describe a clinical case of a patient (man, 49 years old, director of the private agriculture company) diagnosed with two melanomas and one other skin cancer within two years. The patient is also suffering from CML, which appeared after recovering from Covid-19 and has been taking the drug Gleevec from the time of leukemia diagnosis (**Figure 1**).

The patient did not experience any sunburns in the past as he was never tanning - before Covid-19 that occurred in 2020, he was healthy, with no chronic diseases. He was working mostly in the office and have always led a healthy lifestyle (sports, sauna, no smoking).

After skin lesion removal in 2022, the patient was not undergoing any pharmacological treatment against skin cancer. He also did not experience any side effects following Gleevec administration. Generally, the patient report excellent results of treatment and feels good.

During the first year after surgery, the patient did the ultrasound examination of lymph nodes and dermoscopy for skin lesions once every 3 months (later - once every 6 months). He also did regular blood tests for leukemia. No recurrence was reported

### 2.1 Anamnesis morbi

According to the patient’s relation, in 2020, after SARS-CoV-2 infection, a basal cell carcinoma appeared on his neck. In 2021, 3 to 6 months before examination, a dark spot appeared on the nose. Earlier, it was a light brown mole, that the patient remembers from adolescence. Regarding the neoplasm on the back - the patient does not remember the time of its first appearance.

### 2.2 Anamnesis vitae

The course of Covid-19 was severe - the patient experienced 15 days of high fever (39,5-40,0 Celsius degrees) and got Fraxiparine and 3 different intramuscular antibiotics during 20 days. The lung damage according to computed tomography was 25-50%.

3 months after recovering from Covid-19 at the beginning of 2020, chronic myeloid leukemia (2023 ICD-10-CM Diagnosis Code C92.1) was diagnosed and the drug Gleevec was prescribed (400 mg per day), preceded by one month of Hydroxycarbamide administration.

Later in 2020, the patient was also diagnosed with basal cell carcinoma on the neck, which was removed in January 2022. In 2022, melanoma was detected on the nose (the patient noticed the changes 3-6 months before the examination) and on the upper third of the back (he does not remember when it appeared).

### 2.3 Anamnesis familiae

The patient’s mother died in 1979, possibly from leukemia.

### 2.4 Dermatoscopy results

**Figure 2:**
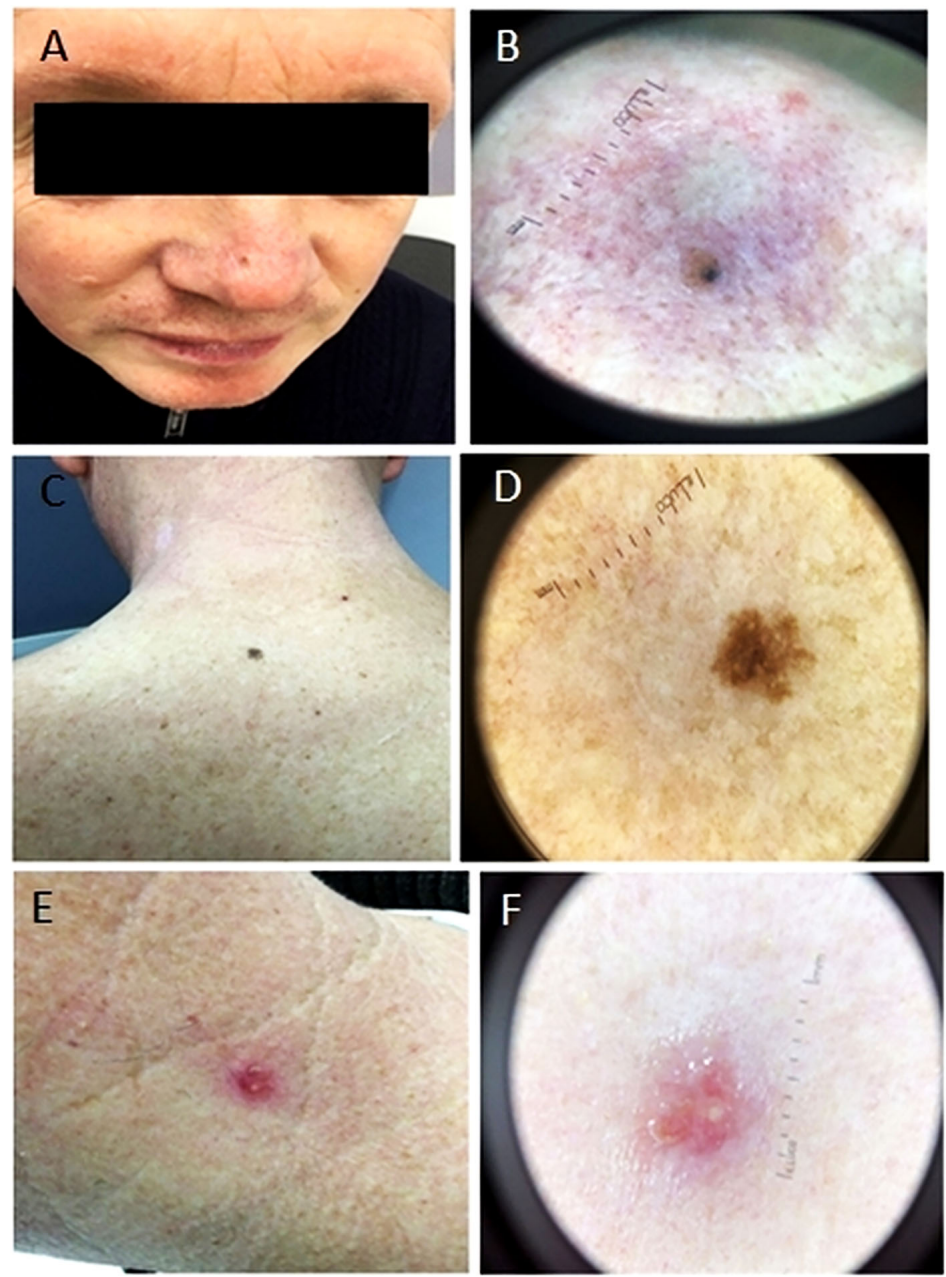
Photographs showing lesion localization on the nose, back and neck (1A, 1C and 1E respectively) and their macro photographs (1B, 1D and 1F respectively). **(A, B)** Light brown macula with an area characterized by dark blue spots. **(C, D)** A macula of uneven color from light brown to dark brown, with uneven edges. **(E, F)** A bright pink papule, round in shape, with a serous crust on the surface.

Nose (**Figures 2A, B**): On the homogeneous light brown background, atypical dark brown globules are visible, located on the periphery of the lesion, at 12 o’clock and 1 o’clock, and closer to the center of the neoplasm at 6 o’clock. Moreover, at 10 o’clock there is a dark blue spot, 1 mm in diameter, around which there is a blue halo, whereas at 8 o’clock there is the area of dark brown unformed pigment.

Back (**Figures 2C, D**): A neoplasm is of irregular shape and polymorphic color from light brown to dark brown in the central part, 6 mm in size. There is an area of irregular dark brown pigment in the center and single atypical globules, on the periphery an atypical pigment grid is visible.

Neck (**Figures 2E, F**): On the left side of the neck there is an injured papule of dark pink color, with tree-shaped vessels and a light yellow crust on the surface, 7 mm in size.

After patient’s physical examination and anamnestic data analysis, the following clinical diagnoses were made:

- Nose - Neoplasm of the skin (possibly combined nevus or atypical nevus) of the nose
- Back - Atypical melanocytic nevus of the skin on the left third of the back
- Neck – Possibly basal cell carcinoma of the skin on the left of the neck

### 2.5 Histopathology

The removed skin lesions underwent a pathohistological examination and (in case of nose and back) also immunohistochemical (IHC) examination. After tumor removal, no recurrence was reported.

#### 2.5.1 Lesion on the nose 

Despite the large number of analyzed slides, the histological picture remains ambiguous and, taking into account the lesion’s localization and the patient’s age, the morphology is most characteristic of Lentigo malignant melanoma (pT2aNxMx V0 L0 Pn0 R0; Breslow Stage II; Сlark lvl IV, TiL - Absent). For the final diagnosis and evaluation of the melanocyte population, further IHC studies are recommended to assess 4 markers: preferentially expressed antigen in melanoma (PRAME), SRY-box transcription factor 10 (SOX-10), Human Melanoma Black-45 (HMB-45) and Ki-67.

**Figure 3:**
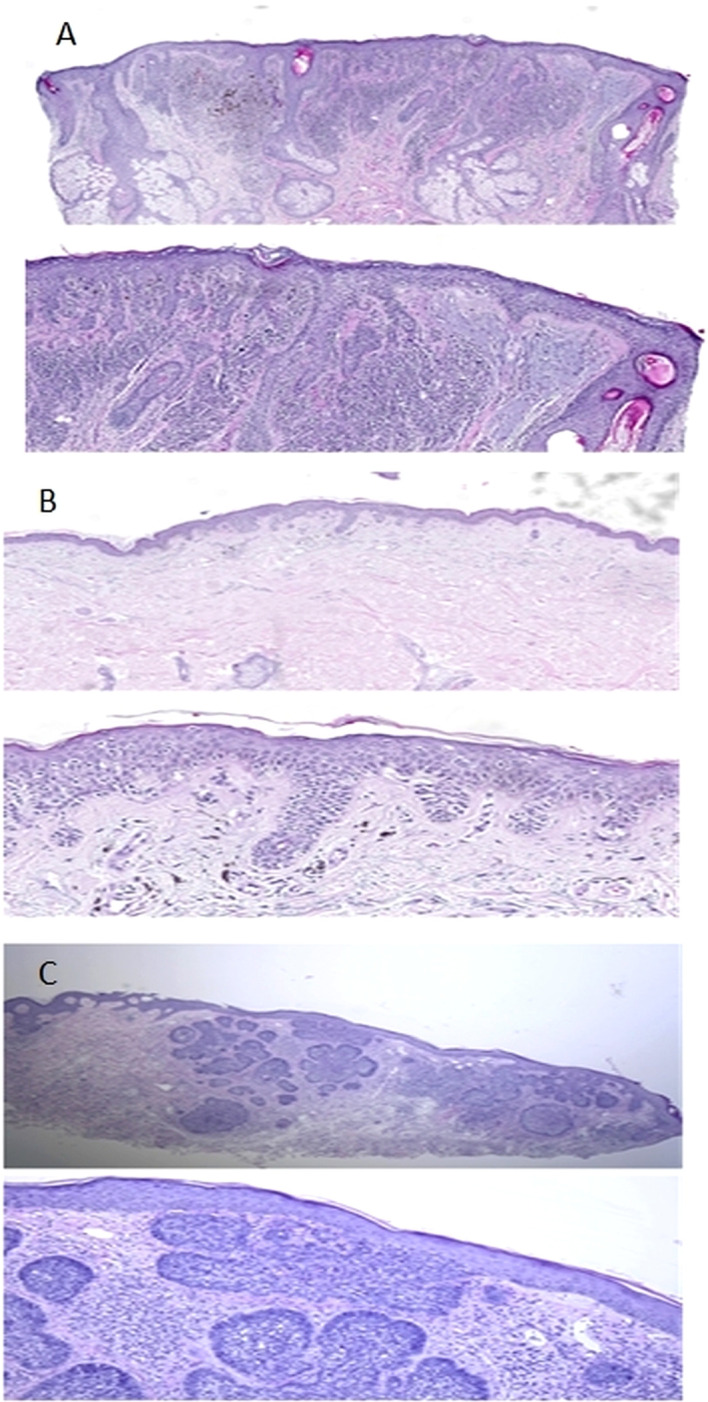
Microphotographs of fragments of skin on the nose, back and neck (3A, 3B and 3C respectively). **(A)** Punch biopsy of the skin from the nose area. A series of sections show a lentiginous distribution of atypical melanocytes (there are also areas of their perifollicular distribution) and an intradermal component represented by 2 cell populations. The first one contains monomorphic melanocytes (Type A) and the second one is represented by polymorphic melanocytes without maturation. Moreover, solaris elastosis (grade 3) is visible in the dermis. Diagnosis: Lentigo maligna melanoma (pT2aNxMx V0 L0 Pn0 R0; Breslow Stage II; Сlark lvl IV, TiL – Absent. **(B)** At the level of the basal layer of the epidermis, there are groups of atypical melanocytes with visible phenomena of pagetoid spread. The invasion into the dermis is observed after SOX-10 staining. According to the results of PRAME staining, a nuclear reaction is observed in all atypical melanocytes. No solar elastosis was found. Diagnosis: Low-CSD melanoma (superficial spreading melanoma) ICD-O code 8743/3, pT1аNxMx V0 L0 Pn0 R0. Clarks Level 2; Breslow stage I (0,168 мм); TiL - Absent. **(C)** Epidermis is without pathological changes. The focal proliferation of atypical basaloid cells with the formation of nodular structures is detected in the dermis. No solar elastosis was found. Diagnosis: Nodular basal cell carcinoma of skin min pT1R1.

**IHC** – Considering the structure of the formation after routine hematoxylin&eosine (H&E) staining and the results of the image-guided coring (IGC) study, there is a growth of lentigo-malignant melanoma on the background of a melanocytic nevus (stage II - in accordance with previous conclusions) (**Figure 3A**).

#### 2.5.2 Lesion on the back

The morphological picture corresponds to atypical melanocytic proliferation with an uncertain potential for malignancy and superficial spread - SAMPUS (Superficial Atypical Melanocytic Proliferations of Unknown Significance). Morphological changes are ambiguous and can correspond to both MIS (Melanoma *in situ* with spitzoid features) and spitzoid nevus. For the final diagnosis, further ICH studies on SOX-10, Melan A and PRAME markers are recommended. SOX-10.

**IHC** - Results of routine examination (H&E) and IGC show, that there is a melanoma that arose on the skin without former significant sun exposure with predominant superficial spreading. It corresponds to diagnosis of low-CSD melanoma (superficial spreading melanoma) ICD-O code 8743/3, pT1аNxMx V0 L0 Pn0 R0. Clarks Level 2; Breslow stage I (0.168 mm); TiL - Absent (**Figure 3B**).

#### 2.5.3 Lesion on the neck 

Atypical basaloid cells with formation of nodular structures without solar elastosis corresponds to diagnosis of nodular basal cell carcinoma of the skin (Nodular basal cell carcinoma ICD-O code 8097/3) min pT1NxMx R1 (**Figure 3C**).

## 3 Discussion

Description of our clinical case indicates, that the primary disease was Covid-19, soon after which the patient was diagnosed with leukemia. Although anamnesis familiae suggests possible genetic predisposition of the patient to leukemia, it is reasonable to suppose that SARS-CoV-2 infection can create a microenvironment that is ideal for neoplastic transformation and malignant conversion. Indeed, epidemiological studies suggest that chronic inflammation may be linked to 15–20% of cancer deaths worldwide (10, 11). This assumption is also confirmed by some case studies concerning CML in children – they were admitted to the hospital with leukemia shortly after recovering from Covid (12).

Moreover, Costa B.A. et al. (3) report three cases of leukemic process development in previously healthy young patients (aged 31-35), with correct blood analysis results, in which the clinical manifestation of leukemia appeared approximately 2-3 months after SARS-CoV-2 infection. Taking into account the rapid development of cytopenia after recovering from Covid-19, the authors suggest that SARS-Cov-2 infection plays a role in leukemogenesis, mainly by provoking the imbalance of the renin-angiotensin system (RAS). The virus interacts with the RAS by binding to angiotensin-converting enzyme 2 (ACE2), which is believed to be involved in tumor hematopoiesis (13) (14). ACE2 tightly binds SARS-CoV-2 receptor-binding domain (RBD) which allows the virus to penetrate cells of various tissues, including bone marrow (15). This causes downregulation of ACE2, which leads to excessive angiotensin-II concentration and resulting immune cell activation and lung injury (16) (17). Moreover, non-physiological levels of angiotensin-II can lead to a leukemic transformation of bone marrow hematopoietic cells. The role of the RAS system in tumor growth is also confirmed by other researchers (18, 19).

There are also other potential mechanisms of cancer development following SARS-CoV-2 infection In particular, Covid-19 has been associated with T-cell exhaustion and activation of oncogenic pathways, including JAK-STAT, MAPK, and NF-kB (20, 21). Moreover, in 2016 Ma-Lauer et al. reported, that the part of coronavirus nonstructural protein (Nsp3) increases CHY zinc-finger domain-containing 1 (RCHY1)-mediated p53 degradation associated with apoptosis (22).

In our case, it was after Covid-19 that the patient was diagnosed with chronic myeloid leukemia, two melanomas, and one skin cancer within one year. Therefore, the possibility of the influence of Covid-19 on carcinogenesis is very high and requires further research.

There are also some data indicating, that malignant melanoma itself could be associated with the SARS-CoV-2 virus (4). Following SARS-CoV-2 infection we often observe upregulation of proinflammatory molecules like S100 calcium-binding protein B (S100B), high-mobility group box-1 (HMGB1), osteopontin (OPN), tumor necrosis factor-alpha (TNF-α), and other cytokines that promote hyperinflammation. The same immunoregulatory proteins that contribute to the Covid-19 “cytokine storm” are also produced by melanoma cells and various other cancers promoting tumorigenesis (4). The authors suggest, that previous SARS-CoV-2 infection may provoke malignant tumor *de novo* development, its aggressive growth, or recurrence. Moreover, high concentrations of Covid-19-associated pro-inflammatory proteins like TNF-α, interleukin (IL)-1α, IL-1β, IL-6, and ferritin increase depigmentation or hypopigmentation of the skin by interfering with tyrosinase synthesis, which is an enzyme catalyzing the rate-limiting pigmentation step. All these molecular events can also cause flare-ups of chronic inflammatory skin diseases and appearance of adverse skin reactions (like for example urticaria, pernio-like reactions of the acral sites, herpes zoster, morbilliform rashes, diffuse hair loss) which can potentially lead to the development of different non-melanoma skin cancers (8). To sum up, the ability of SARS-CoV-2 to increase the expression of proinflammatory and oncogenic molecules may have a pro-carcinogenic effect and may contribute to the emergence or development of various types of cancers, especially in patients who suffered from severe form of Covid-19 (4).

It is also worth mentioning, that although the exact methods of Covid-19 treatment in case of our patient remain unknown, there are some concerns that Molnupiravir (antiviral drug) can potentially cause cancerous mutations or birth defects (23).

It is already known that CML is a myeloproliferative neoplasm, the mechanism of which is the clonal proliferation of tumor-altered polypotent stem cells of the bone marrow. Thus, some researchers claim, that blood cancers like CML may trigger or may be associated with the occurrence of other malignant neoplasias (24). The exact etiological factors of CML development remain unclear, but it is known, that as a result of translocation of the 9th and 22nd chromosome’s long arms, the Philadelphia chromosome (Ph) arises. Consequently, the BCR and ABL1 genes merge forming the chimeric BCR-ABL1 gene – its product (the bcr-abl protein) exhibits constant tyrosine kinase activity, which leads to increased proliferation of the bone marrow stem cells, inhibition of apoptosis and impaired adhesion of leukemic cells to the bone marrow stroma. As a consequence, the acceleration phase and blast crisis occur, which are characterized by the accumulation of cytogenetic aberrations, resistance to treatment and an unfavorable prognosis. That is why leukemia may increase the risk of other tumors development (7). However, authors indicate, that the higher incidence of secondary malignancies in CML patients can result from many additional factors like for example: genetic predispositions, exposure to risk factors (common for CML and other cancers), the CML treatment itself and the increased medical surveillance in first years after diagnosis (7).

As far as genetic predispositions are concerned, there are some studies highlighting the role of genetic pleiotropy in the pathogenesis of leukemia and non-melanoma skin cancer. It was showed, that a single pleiotropic locus (6p25.3) is responsible for the observed association between susceptibility to squamous cell carcinoma and increased chronic lymphocytic leukemia risk. Mentioned study also proves, that genetic susceptibility to chronic leukemia increases basal cell carcinoma risk (25).

In case of our patient, there can be some genetic predispositions to cancers, especially to CML. On the other hand, it is difficult to draw evident conclusions about the direct/indirect effect of existing CML on other tumors formation, due to the previous Covid-19 disease, which can directly provoke carcinogenesis or create favorable conditions for tumor appearance. However, there are some data about melanoma and non-melanoma skin cancers occurrence during the course of hairy cell leukemia (26), so the hypothesis of leukemia-associated cancers is highly probable and requires further investigation.

Interestingly, our patient was treated with tyrosine kinase inhibitor (TKI) Gleevec (Imatinib mesylate) for a long time. According to the data from Occupational Safety and Health Administration (OSHA) (27). Gleevec inhibits the BCR-ABL tyrosine kinase, which is a constitutive, abnormal tyrosine kinase coded by the gene located on the Philadelphia chromosome in CML patients. This medication is also used to treat other types of cancers (such as acute lymphoblastic leukemia, gastrointestinal stromal tumors, and myelodysplastic/myeloproliferative diseases). It inhibits proliferation and induces apoptosis of BCR-ABL positive cell lines as well as of fresh leukemic cells in case of Philadelphia chromosome-positive CML (27). Gleevec is also an inhibitor of tyrosine kinase receptors for platelet-derived growth factor (PDGF) and stem cell factor (SCF), so it inhibits PDGF- and SCF-mediated cellular events. *In vitro*, imatinib decreases proliferation rate and induces apoptosis in GIST cells, which express c-Kit mutation.

Despite the positive effect of Gleevec continuous administration (it prolonged patient’s survival) there are some concerns regarding its possible impact on secondary malignancies development (28). A long-term observational, population stidy of CML patients showed, that risk of secondary malignancies was increased (29, 30). However, it was not increased in case-control studies of CML patients treated with TKI (31, 32). So far, it is impossible to select patients in the observed populations for the effect of specific CML treatment on the risk of secondary malignancies (6). Therefore, the question if secondary malignancies occur after long exposure to TKI remains unanswered.

The main limitation of our study is a short follow-up period (lack of information about long-term outcome) and limited data about severity of CML and antibiotics used during Covid-19 treatment. Our patient was one of the first hospitalized patients during pandemic, so there were no proven, permanent treatment protocols at that time.

In the future, it would be advisable to perform long-term follow-up studies on large cohorts of CML patients treated with TKIs. As large cancer registries often do not include complete data on treatment methods, it would be helpful to create a base of CML patients taking part in case-control studies.

### 3.1 Conclusions

Numerous studies, ranging from clinical investigations to molecular analyses, have highlighted a strong contribution of inflammation, especially Covid-19 associated uncontrollable cytokine storms, to the development of various types of skin lesions (33) as well as leukemia, melanoma, and other cancers. Moreover, it is proven, that inflammation in the tumor microenvironment not only creates favorable conditions for melanoma and other cancer progressions, but also inhibits antitumor immunity from boosting in the course of cancer immunotherapy. Our clinical case highlights the necessity to consider the regular oncological control of patients after recovering from Covid-19. Moreover, in the case of leukemia, doctors should recommend a patient’s careful, dermatological examinations, as there is a possibility of secondary skin tumors. At the same time, so far published data show, that TKIs (like Gleevec) are unlikely to be the direct cause of other cancers, while SARS-CoV-2 and other viruses are supposed to have a direct and indirect influence on carcinogenesis.

## Data availability statement

The original contributions presented in the study are included in the article/supplementary material. Further inquiries can be directed to the corresponding author.

## Ethics statement

Written informed consent was obtained from the individual(s) for the publication of any potentially identifiable images or data included in this article.

## Author contributions

OBo: Conceptualization, Investigation, Project administration, Supervision, Writing – original draft. ER: Formal Analysis, Investigation, Writing – review & editing. RH: Data curation, Visualization, Writing – review & editing. CB: Data curation, Visualization, Writing – review & editing. OBe: Data curation, Investigation, Visualization, Writing – review & editing.
